# Increased level of circulating cell-free mitochondrial DNA due to a single bout of strenuous physical exercise

**DOI:** 10.1007/s00421-020-04330-8

**Published:** 2020-02-22

**Authors:** Lars Ohlsson, Anna Hall, Hanne Lindahl, Ravi Danielsson, Anna Gustafsson, Eva Lavant, Lennart Ljunggren

**Affiliations:** 1 Department of Biomedical Science, Malmö University, Malmö, Sweden; 2 Department of Clinical Chemistry, SUS, Region Skane, Malmö, Sweden; 3 Department of Clinical Pathology, SUS, Region Skane, Malmö, Sweden

**Keywords:** Cell-free mitochondrial DNA, Exercise, Inflammatory response, Leukocytes, Soluble urokinase plasminogen activator receptor

## Abstract

**Purpose:**

Physical exercise is reported to affect the immune response in various ways. Thus, the levels of pro-inflammatory cytokines as well as the abundance of circulating leukocytes are changed. In this study, the occurence of circulating cell-free mitochondrial DNA (cfmtDNA) and nuclear DNA (nDNA) was investigated in connection with a single bout of strenuous physical exercise.

**Methods:**

Healthy volunteers performed a controlled ergo-spirometry cycle test and venous blood samples were taken at different time-points to analyze the concentration of blood components before, during and after the test. The number of circulating leukocytes was measured, as well as secretion of the soluble urokinase activator receptor (suPAR).

**Results:**

Cf-mtDNA significantly increased during exercise, compared to baseline values and after 30 and 90 min of rest. Circulating leukocytes increased during exercise, but returned to baseline levels afterwards. Surface expression of the urokinase plasminogen activating receptor (uPAR) on neutrophils decreased significantly during exercise. The concentration of suPAR tended to increase during exercise but only significantly after 90 min of rest.

**Conclusion:**

Increased concentration of cf-mtDNA indicates that cell damage takes place during high intensity training. Hypoxia and tissue damage are likely causes of cf-mtDNA from muscle cells. The levels of cf-mtDNA remain high during the initial rest, due to the decreasing numbers of leukocytes normally clearing the plasma from cf-mtDNA. The increased levels of suPAR further emphasize that strenuous physical exercise causes a reaction similar to inflammation. Further studies are needed to detect the source of increased cf-mtDNA and the corresponding increase of suPAR liberation.

## Introduction

The effect of strenuous physical exercise on the immune system has been studied during the last years. Both acute and chronic training have been investigated, often in connection with various disease syndromes, such as depression or multiple sclerosis (Hallberg et al. 2010; Mikkelsen et al. 2013; Suchanek et al. 2010; Zwetsloot et al. 2014). A single bout of physical activity leads to both cellular and humoral immune responses similar to those caused by infection, sepsis and trauma (Steensberg et al. 2000). The plasma concentration of the pro-inflammatory cytokines (e.g. TNF-α, IL-1β, IL-6 and IFN-γ) as well as acute-phase proteins increase (Boettger et al. 2010; Castellano et al. 2008). However, the degree of immune activation is reported to decrease in persons that perform regular training (Simpson et al. 2015).

Mitochondria are organelles responsible for the synthesis of ATP (Johannsen and Ravussin 2009; Scheffler 2001) and are also responsible for the generation of signaling molecules involved in apoptosis and necrosis (Johannsen and Ravussin 2009). The number of mitochondria per cell depends on its energy requirement, and e.g. muscle cells are equipped with many mitochondria to perform their regular work (Wust et al. 2018). Regular exercise has been shown to increase the number of mitochondria in muscle cells, resulting in an increased ability to synthesize ATP. In case of cellular damage, endogenous cellular substances normally not exposed to the immune system, act as danger-associated molecular patterns (DAMPs), as they are released into the circulation. These molecules are then recognized by different pattern recognition receptors, such as Toll-like receptors (TLRs), on immune cells causing a signaling cascade leading to excretion of antimicrobial substances and pro-inflammatory substances (Zhang et al. 2010a, b). Mitochondrial DNA (mtDNA), released into circulation from mitochondria under stress, has been reported to act as a DAMP, mimicking an ongoing immune-activation, mediating a pro-inflammatory response (Bhagirath et al. 2015; Chiu et al. 2003; Yu 2012). Systemic inflammatory response syndrome (SIRS) is a condition when several pro-inflammatory mediators, due to cellular damage caused by tissue-damage, trauma or hypoxia are released into the blood. Since heavy physical exercise, due to muscle damage, necrosis and oxygen deprivation has been shown to result in immune-activation resembling trauma and sepsis, the contribution of mitochondrial function has to be taken into account. Studies of trauma have revealed that inflammation, neutrophil activation and organ injury can be mediated by cell free mtDNA (cf-mtDNA) activating neutrophils via TLR-9, normally binding bacterial DNA (Zhang et al. 2010a, b).

Another useful biomarker for immune activation is the soluble urokinase plasminogen activating receptor (suPAR) (Thuno et al. 2009) however, only a few studies can be found describing the effect from intense physical activity on suPAR (Gustafsson et al. 2017; Sanchis-Gomar et al. 2013). suPAR is the soluble fragment of the multi-ligand receptor urokinase plasminogen activating receptor (uPAR or CD87). uPAR, expressed on several cell types, including neutrophils, monocytes, and endothelia cells, is involved in both proteolysis, cell-migration, angiogenesis, and inflammation. During the last decades, several studies have been investigating the possible use of suPAR as a biological marker of inflammation, organ damage, and severity of the disease, showing positive correlations between increased suPAR concentrations in both blood, urine and cerebrospinal fluid (Enocsson et al. 2015; Thuno et al. 2009).

During infection and inflammation, as well as during physical exercise, an increased number of circulating leukocytes is observed (Pedersen and Hoffman-Goetz 2000). Leukocytosis during physical exercise is caused by de-margination of leukocytes from the marginal pool caused by increased heart rate and blood flow, as well as by increased concentrations of epinephrine and cortisol (Sanchis-Gomar and Lippi 2014). Neutrophils, dendritic cells and lymphocytes have shown to increase during exercise (Pedersen and Hoffman-Goetz 2000; Suchanek et al. 2010). After exercise, the lymphocytes decrease below base-line but the levels of neutrophils remain increased (Pedersen and Hoffman-Goetz, 2000; Simpson et al. 2015).

The aim of this study was to investigate if a bout of strenuous physical exercise leads to changed levels of circulating cf-mtDNA in plasma and to examine if a correlation between cf-mtDNA levels, suPAR, and total leukocytes could be established. Moreover, a possible correlation of membrane-bound uPAR (CD87) and free suPAR was investigated.

## Materials and methods

Healthy volunteers (*n* = 8) performed a controlled ergo-spirometry cycle test and venous blood samples were taken at different time-points to analyze the concentration of blood components before, during and after the test. The subjects were requested not to eat or drink 2 h before the test, nor drink alcoholic beverages during the day before the test.

### Subjects

Eight non-smoking volunteers [four female and four male, aged 20—61 (38.6 ± 14.4)], with no known history of heart- or lung disease and a normal BMI participated in the cycle test. The actual fitness of the subjects was not known. Ethical approval for the implementation of the study was obtained from the Ethical Council of the Faculty of Health and Society, Malmö University (Ref: HS60-2015/306: 2) and the study was performed, according to the Helsinki declaration (2013). All participants gave their written consent.

### Ergospirometry, samples collection and handling

When the test started the resistance for female participants was set to 30 W on the Ergometer (Monarch Ergomedic 939E, Monark Exercise AB in Vansbro, Sweden) and was subsequently gradually allowed to increase by 10 W/min. Male participants started at 50 W, gradually increased by 15 W/min. Throughout the test, the volunteers were encouraged to keep a steady pace on the pedals (about 60–65 revolutions/min). Blood samples were taken via a peripheral venous catheter (BD Venflon™ Pro Safety and BD Connecta™, Becton Dickinson, Helsingborg, Sweden) at five specific time points (on arrival after 15 min of rest (zero/0-sample), at submaximal- and maximum load, and at 30 and 90 min after the test). After each sampling, the catheter was flushed with 5 mL of physiological saline. Time of sampling at the maximum load took place when the subjects reached exhaustion, indicating that they could not cycle any further. All blood samples were taken while the subjects were sitting, either at the bike or in an upright position. Sub-maximal load was considered to be reached either when the subject had cycled for six minutes at an increasing resistance or when a heart rate of 125 ± 5 beats/min was reached. Blood samples were collected in EDTA vacutainers and placed on ice or at room temperature depending on further analysis. Plasma from blood samples was prepared by centrifugation within one hour of sampling at 4 °C at 2000×*g* for 10 min, and stored at − 80 °C. Correction of plasma volume changes during the test was performed using blood hemoglobin values (Alis et al. 2015).

### Hematology analysis

Determination of leukocyte concentration, differential counts, and hemoglobin concentration was performed on blood from the sampling tube which had been stored at room temperature (between one and five hours). The blood was mixed well and then analyzed with HemoCue® WBC DIFF and HemoCue Hb 201 + instruments (HemoCue AB, Ängelholm, Sweden), following the manufacturer's instructions.

### Flow cytometric analysis

Within three hours from sampling, 100 µL of blood was transferred into 1.5 mL tubes. Labeling of cells was made with the following antibodies from BD Biosciences (Stockholm, Sweden): APC mouse anti-human CD11b, PE mouse anti-human CD87, APC mouse anti-human CD14 and APC mouse-anti human CD3. Corresponding isotype antibodies were used as negative controls. The tubes were incubated at room temperature for 30 min and erythrocytes were lysed using 1 mL FACS Lysing Solution (BD Biosciences, Stockholm, Sweden) for 30 s at room temperature. The cells were then washed with 1 mL PBS (Life Technologies Europe BV, Stockholm, Sweden) with 1% BSA (Sigma-Aldrich Sweden AB, Stockholm, Sweden). The tubes were centrifuged at 1000×*g* for five minutes and the supernatant was discarded and the washing procedure was repeated once more. Finally, the cells were resuspended in 0.5 mL CellFIX (BD Biosciences, Stockholm, Sweden) and protected from light before flow cytometric analysis.

The flow cytometric analysis was performed on an Accuri C6 (BD Biosciences, Stockholm, Sweden) with the associated software CFlow Plus (BD Biosciences, Stockholm, Sweden). For each sample, data were collected from 25,000 cells in the total leukocyte gate.

### Analysis of suPAR

Plasma (EDTA) suPAR concentrations were analyzed using a commercially available enzyme immunoassay (suPARnosticTM, Virogates, Copenhagen, Denmark), according to the manufacturer’s instructions. The assay detects all circulating suPAR, including intact and cleaved forms of the receptor.

Samples were analyzed in duplicates and the mean was used for statistical analysis. The detection limit for suPAR was 0.1 ng/mL according to the manufacturer.

### Measurement of circulating cell-free mitochondrial and nuclear DNA

The isolation and quantification of cf-mtDNA/nuclear DNA (nDNA) in plasma samples has previously been described (Lindqvist et al. 2016). Briefly, the thawed plasma samples were centrifuged for 10 min at 10,000×*g* and DNA was isolated from the top 200 µl using the QIAmp 96 DNA Blood Kit (Qiagen, Valencia, CA, USA) according to the manufacturer’s instruction using the blood and body-fluid protocol. The isolated DNA was eluted in 200 µl Tris–EDTA-buffer (TE-buffer) and quantified with a Nanodrop (ND-1000 Spectrophotometer v 3.7.1, Waltham, MA, USA) using spectrophotometric analysis at 260/280 nm. The quantitative analysis of cf-mtDNA and nDNA (NADH dehydrogenase, ND2 and beta-2-microglobulin, (β2M)) was performed using quantitative real-time polymerase chain reaction (qPCR). The PCR reactions were carried out using SYBR Green Technology (Thermo Fisher Scientific, Waltham, MA, USA). Each 20 μl reaction contained 5 μl of template, 1 μL of each primer (10 μM), 10 μL SYBR MIX (2 × Sensifast, Bioline, London, UK) and 3 μL of nuclease-free water. Each reaction was run in triplicate on a LC480 LightCycler from Roche, Mannheim, Germany) using the following program: Initial denaturation at 95 °C for 10 min, 45 cycles consisting of 95 °C for 10 s, 65 °C for 10 s and 72 °C for 11 s. The program was terminated with a melting curve analysis measuring fluorescence continuously from 60 to 97 °C.

Serial dilutions (dilution factor 10) in eight steps of the purified PCR products, starting with 56 pg/µL (ND2) or 4L pg/µl (β2M) were used to create standard curves. The obtained crossing-point values from the unknown samples were compared with the standard curve, and the corresponding number of mitochondrial or nuclear units was calculated. Calculations were carried out as described in Lindquist et al. (2016).

### Statistics

Data are presented as mean values ± SD. Differences at various time points compared to base-line has been calculated using Mann–Whitney *U* test. Correlation was calculated using Spearman's test. Significant levels were *P < 0.05, **P < 0.01 and ***P < 0.001.

## Results

During this study, the effect of strenuous physical exercise on circulation of various leukocytes and inflammatory markers, such as circulating cf-mtDNA and suPAR were measured. Maximal heart rate of the participants was reached at individual time points ranging from 16.5 to 25.5 min, with a median value of 20.5 min (Table 1).

**Table 1:** The primers (Life Technologies, Pailsey, UK) used for PCR amplification of cf-mtDNA and nDNA, respectively

Gene	Primer forward	Primer reverse	Access nr
ND2	CACACTCATCACAGCGCTAA	GGATTATGGATGCGGTTGCT	KJ676545
β2M	TGTTCCTGCTGGGTAGCTCT	CCTCCATGATGCTGCTTACA	M17987

### Circulating cell-free mtDNA (cf-mtDNA)

**Fig. 1:**
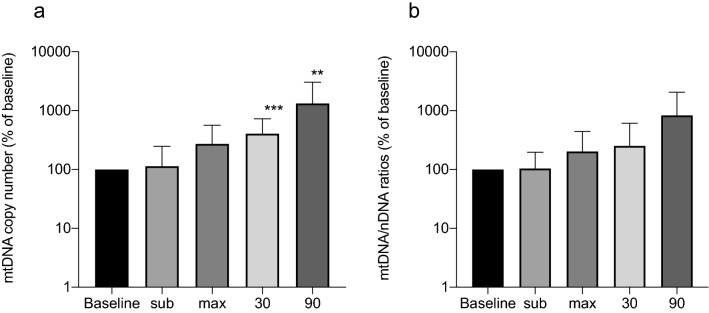
Relative change of circulating cf-mtDNA compared to baseline at various time points during and after exercise (**a**) or relative change of rations between mtDNA/nDNA compared to baseline (**b**)

The presence of cf-mtDNA in plasma increased in all participants during exercise. The kinetics for the presence of cf-mtDNA and cf-nDNA resp. indicate that the amount of cf-mtDNA increased more during rest while cf-nDNA mainly increased closer to maximal workload (Table 2). However, a significant increase (*P* < 0.05) was only observed 30 and 90 min after maximal work-load. Although there was an individual difference of the absolute levels of cf-mtDNA units, the relative difference between time point P-0 (before the exercise) and all other time points was substantial. Even after 90 min of rest from the physical test, the level was more than 25 times increased (Fig. 1a). The ratios between cf-mtDNA and cf-nDNA increase during and after exercise, however, not significantly, compared to baseline values (Fig. 1b).

**Table 2:** Amount of DNA during different time points of exercise. Results are given as mean values ± SD

DNA origin	P-0	P-sub	P-max	P-30	P-90
cf-mtDNA, copies/L × 10^6^	22.2 ± 26.3	39.7 ± 94.0	26.5 ± 21.0	90.8 ± 153.8	228.9 ± 483
cf-nDNA, copies/L × 10^6^	28.22 ± 58.8	15.8 ± 29.7	46.1 ± 69.6	49.6 ± 50.6	39.0 ± 52.6

### Circulating leukocytes

**Fig. 2:**
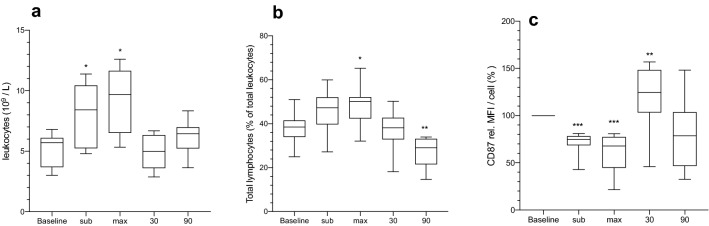
Circulating leukocytes (10^9^/L) in blood (**a**), total lymphocytes (% of leukocytes) (**b**) and mean fluorescence intensity (MFI) from CD87 (% of base-line) of neutrophils (**c**). *P < 0.05, **P < 0.01 and ***P < 0.001 using Mann–Whitney *U* test

The total number of blood leukocytes increased significantly (*P* = 0.0013) from 5.2 × 10^9^/L to a maximal of 9.0 × 10^9^/L during maximal workload but returned to baseline values after 90 min of rest (Fig. 2a). Circulating lymphocytes were significantly increased at maximal workload, compared to baseline, but returned to baseline after 30 min of rest. 90 min of rest resulted in a significant decrease of lymphocytes, compared to baseline (Fig. 2b). The number of circulating monocytes and neutrophils increased significantly from baseline until maximal workload was reached (data not shown).

The mean fluorescent intensity from CD87 on neutrophils showed a significant decrease during sub-maximal and maximal work-load, but an increase after 30 min of rest (Fig. 2c).

### Concentration of plasma suPAR.

**Fig. 3:**
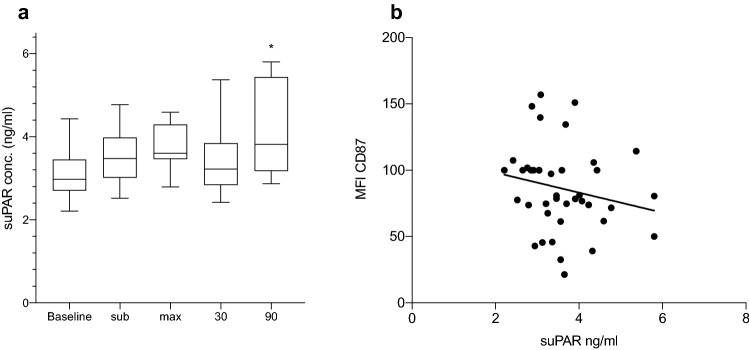
Concentration of suPAR during all time points (**a**) and correlation between suPAR and MFI from CD87 (Spearman *r* =− 0.25) (**b**). *P < 0.05 using Mann–Whitney *U* test

The level of plasma suPAR was measured during all time points of the exercise showing a biphasic pattern. During the initial part of work-load, increased concentrations could be seen, however, after 30 min rest the concentration dropped but reached its highest concentration 90 min after maximal effort (Fig. 3a). Furthermore, the concentration of suPAR and MFI from CD87 on granulocytes showed an inversed relation, however, not significant (*P* = 0.0624) (Fig. 3b). No correlation between the levels of suPAR and the number of neutrophils was found.

## Discussion

This study was performed to investigate if a single bout of strenuous physical exercise affects the concentration of cf-mtDNA and suPAR in plasma. Furthermore, the concentration of total leukocytes was measured during the study, together with the presence of membrane-bound uPAR on the different leukocytes.

The results clearly show that the concentration of cf-mtDNA was dynamically affected during the measurements. At sub-maximal and maximal work-load the concentration was mildly increased but a significant increase of concentrations could be seen after both 30 and 90 min of rest, compared to baseline levels. These results indicate that strenuous physical exercise induces some kind of cellular damage, leading to necrosis and the liberation of mitochondrial DNA. The results from the present study regarding cf-mtDNA differ from other studies showing decreased values of cf-mtDNA (Beiter et al. 2011; Helmig et al. 2015). In one extensive study, repeated bouts of exhaustive exercise were carried out, giving rise to large increases of both cf-mtDNA but especially cf-nDNA (Stawski et al. 2017). A possible reason for the increased levels of cf-DNA, both nuclear- and mitochondrial origin, could be DNA liberation from neutrophils. Liberation of DNA from neutrophils, forming neutrophil-extracellular traps (NET) have been shown to take place rapidly after stimulation (Yousefi et al. 2009). Possible reasons for the contradicting results could be technical differences during sample treatment and DNA-isolation. Slightly different protocols are in use, with differences of centrifugation speed and time, among other things (Dantham et al. 2016; Kumar et al. 2017). A reason for the raised values of the ratios between cf-mtDNA and n-DNA could be the fact that the primers targeting b2M do not bind to shorter fragments of damaged nuclear DNA (Jiang and Lo 2016).

Previous studies have shown that tissue damage occurs during physical exercise (Zwetsloot et al. 2014). Muscle cells, heavily loaded during physical exercise, are richly equipped with mitochondria. Hypoxia and tissue damage, due to over-load, are likely causes for the liberation of cf-mtDNA from these cells (Zhang et al. 2010a, b).

In a recent study also investigating the release of cf-mtDNA, another more extended exercise regime was used. The time of a tread-mill run was set to 90 min but only 60% of VO_2_ was used. During this weaker bout of physical work, a decreased level of cf-mtDNA was registered (Shockett et al. 2016), indicating that different grades of physical labour have different effects. Short term hard labour probably causes tissue damages, reminding of trauma, while prolonged medium exercise causes an increase of circulating neutrophils, absorbing liberated cf-mtDNA, resulting in the observed decrease. The samples from the different participants of the present study showed a substantial individual variation. According to previous studies, the concentration of cf-mtDNA is influenced by several factors, such as age and health status (Pinti et al. 2014). The amount of cf-mtDNA has been reported to increase significantly after 50 years of age, however, with large individual differences. Age is an important parameter, probably due to a gradual decrease of muscle mass, leading to increased necrosis and liberation of endogenous substances, including mtDNA (Mikkelsen et al. 2013). Increased levels of cf-mtDNA in plasma could also be a result of decreased ability to clear it from the circulation (Kung et al. 2012). The mechanisms of clearing of cf-mtDNA have been discussed and studied by others and circulating neutrophils have been suggested to bind cf-mtDNA to TLR's acting as a sink (Shockett et al. 2016). Another theory is that increase of plasma DNase I activity, measured directly after exhaustive short-term exercise, could help restoring levels to base-line values (Beiter et al. 2011). Recently, it was reported that a sequence of DNA, targeted by the primers for the ND2-gene in the mitochondrial genome, is present on chromosome 1 in the human nuclear genome (NCBI Gene ID: 100652939). However, during our previous study of cf mtDNA, another primer-pair, targeting the ND1 gene in the mitochondrial genome, both pairs of primers were found to correlate to a high degree (Lindqvist et al. 2018).

An increased level of suPAR could be seen, during the exercise and the following rest, however, suPAR levels were only significantly raised 90 min after maximal work-load (Fig. 3a). Plasma concentrations of less than 4 ng/mL are considered as normal, while levels above 6 ng/mL may indicate illnesses such as cancer, diabetes or cardiovascular disease (Eugen-Olsen et al. 2010; Gustafsson et al. 2017; Kofoed et al. 2008). The cell-surface expression of the membrane-bound variant, uPAR, on the neutrophils was decreased during the same time, giving an almost inverted expression pattern, compared to suPAR (Fig. 3b). The negative correlation, although not significant, between suPAR and cell-surface expression of uPAR, measured as mean-fluorescent intensity (MFI) on granulocytes, supports the results of the study. The changes of suPAR-expression seen in this study are similar as during a study performed on patients suffering from major depressive disorder (MDD) (Gustafsson et al. 2017). An almost identical pattern of plasma-suPAR was found during that study, indicating that a single bout of strenuous physical exercise has a substantial effect on suPAR levels. The rather immediate increase of suPAR and decrease of uPAR fits well with the theory that uPAR is cleaved by a mixture of activated proteases and acute-phase proteins (Thuno et al. 2009). The concentration of suPAR measured by Sanchis-Gomar et al. (2014) before and after a football match was not reported to change, however, the subjects were all athletes, probably accustomed to physical exercise. The blood samples after the match were, however, taken 12 h after the match was finished. In a recent study, including 80 participants and 53 controls, it was reported that mild physical training, during a five-month period of time, did not have any effect on suPAR-levels (Rohde et al. 2018). On the other hand, a healthy lifestyle has been shown to significantly affect the levels of suPAR (Haupt et al. 2019) with higher concentrations measured in smokers and physically inactive participants. One interpretation of the different results is that suPAR levels indicate an acute inflammatory response, as mimicked during heavy physical exercise, but also a low-grade on-going inflammation, manifested as a result of unhealthy and sedentary lifestyle.

The expression of uPAR has been found on several blood leukocytes, including granulocytes, monocytes and activated T-lymphocytes (Koch et al. 2014; Thuno et al. 2009). The leukocytosis, resulting from de-margination of cells during heavy exercise, probably contributes to the increase of suPAR, since the levels of suPAR and total leukocytes have a similar concentration profile. Another explanation to the increase of suPAR could be a possible microbial translocation across the gut, resulting in the occurence of lipopolysaccharide (LPS) (Brenchley et al. 2006; Ng et al. 2008) in connection with physical exercise.

Several different proteases have been demonstrated to cleave uPAR in the linker region, including the urokinase plasminogen activator (uPA) itself, plasmin, chymotrypsin, and various MMPs (Andolfo et al. 2002). Increased plasma levels of uPA have previously been shown to result from physical exercise (Dooijewaard et al. 1991). When uPA is bound to uPAR, the receptor is cleaved, and suPAR, the soluble form of uPAR, is released. Since it has also been shown that heavy exercise can contribute to elevated levels of metalloprotease-9 (MMP-9) (Rullman et al. 2013), this protease could also be involved in the cleavage of uPAR leading to suPAR production.

One limitation of the study is the lack of blood-samples at later time-points. It would be interesting to measure the levels of both cf-mtDNA and suPAR during the following day and compare that to the leukocyte concentration.

A further limitation of the study is the low number of participants, making excessive conclusions from the results difficult. However, the aim of the study was reached, and was in line with the study by Stawski et al. (2017), encouraging further examinations of the effect from exercise on the immune response and the use of cf-mtDNA and suPAR in measurements. The increased levels of plasma IL-6, measured in connection to extensive exercise (Ullum et al. 1994), further strengthens the results indicating that exercise induces an inflammatory reaction manifested in several ways. Further studies will have to be performed to clarify the mechanisms involved.

## Data Availability

The datasets generated during and/or analysed during the current study are available from the corresponding author on reasonable request.

## Abbreviations

ATP: Adenosine-tri-phosphate
β2M: Beta-2 microglobulin
BSA: Bovine serum albumin
cf-mtNDA: Cell-free mitochondrial DNA
cf-nDNA: Cell-free nuclear DNA
DAMP: Danger-associated molecular pattern
EDTA: Ethylenediaminetetraacetic acid
IFN-γ: Interferon-gamma
IL-1β: Interleukin 1-beta
IL-6: Interleukin-6
LPS: Lipopolysaccharide
MDD: Major depressive disorder
MFI: Mean fluorescent intensity
MMP: Matrix metalloproteinase
mtDNA: Mitochondrial DNA
ND1: NADH dehydrogenase 1
ND2: NADH dehydrogenase 2
NET: Neutrophil extracellular traps
PBS: Phosphate-buffered saline
qPCR: Quantitative polymerase chain reaction
SIRS: Systemic inflammatory response syndrome
suPAR: Soluble urokinase-type plasminogen activator receptor
TLR: Toll-like receptor
TNF-α: Tumor necrosis factor-alpha
uPAR: Urokinase-type plasminogen activator receptor
